# Modeling a COVID-19 Vaccination Campaign in the State of Madhya Pradesh, India

**DOI:** 10.1007/s40171-022-00326-9

**Published:** 2022-12-14

**Authors:** Priyanka Das, Santosh Shukla, Ashwin Bhagwat, Saurabh Purohit, Sanjay Dhir, Sushil, Harkabir Singh Jandu, Meenal Kukreja, Nitin Kothari, Saurabh Sharma, Shyamashree Das, Gunjan Taneja, Raj Shankar Ghosh

**Affiliations:** 1 National Health Mission, Department of Health and Family Welfare, Government of Madhya Pradesh, Bhopal, India; 2 Department of Management Studies, Indian Institute of Technology Delhi, New Delhi, India; 3 Clinton Health Access Initiative, New Delhi, India; 4 Bill & Melinda Gates Foundation, New Delhi, India

**Keywords:** Challenges, COVID-19 vaccination, India, Innovation, System dynamics

## Abstract

COVID-19 vaccination campaigns have been launched across the globe to mitigate the impact of the COVID-19 disease. However, COVID-19 vaccination campaigns (Vaccination campaigns are the complete process of COVID-19 vaccination campaigns beyond just administrating vaccines.) are a complex multi-stakeholder process, and therefore, it is important to understand the key components and drivers of vaccination campaigns to help devise strategies to increase vaccination coverage for existing and future vaccination efforts. A system dynamics modeling approach was used to trace the vaccination campaign for the Indian state of Madhya Pradesh. The results identified the key stakeholders of the vaccination campaign and their inter-linkages, and the diverse perspectives of stakeholders of vaccination campaigns in Madhya Pradesh were collated in a structured format. Further, system dynamics models were developed to capture all aspects of the vaccination campaign in Madhya Pradesh, including the challenges and innovations. The outcomes of the study can assist academicians, practitioners, and policymakers develop vaccination programs at sub-national, national, and global levels. They will guide decision-makers to take preemptive measures to mitigate possible risks and challenges and provide improved services as part of vaccination campaigns.

**Supplementary Information:**

The online version contains supplementary material available at 10.1007/s40171-022-00326-9.

## Introduction

COVID-19 is a contagious and infectious disease caused by the SARS-CoV-2 virus. On March 11, 2020, the World Health Organization (WHO) declared it a pandemic (Ghebreyesus, 2020), and as of May 4, 2022, the total number of confirmed cases of COVID-19 has reached 513 million and has accounted for more than six million deaths worldwide. In India, the number of confirmed cases is now more than 43 million (WHO Dashboard, 2022). To mitigate the impact of COVID-19, a large part of the population needs to be vaccinated (Bloom et al., 2020), and vaccination campaigns were launched across the globe in 2020 and 2021. Strengthening health systems for vaccine delivery before and during mass vaccination campaigns and encouraging the public to be vaccinated, maintain clear communication, and dispel the myths related to vaccines are important decision-making areas in vaccination campaigns.

The Government of India launched India’s COVID-19 vaccination program on January 16, 2021 (Boye, 2021). In January 2021, the COVID-19 vaccination campaign was launched for healthcare workers and subsequently for frontline workers and citizens over 45 years in phases between February–April 2021. Then, from May 2021 onwards, vaccination was allowed for people over 18 years (MoHFW, 2021a). As of May 5, 2022, 1.90 billion doses have been administered. This includes more than 867 million fully vaccinated people, approximately one billion people who have received one dose, and more than 2.8 million people over 60 years, people with comorbidities, and healthcare and frontline workers who have received precautionary doses (CoWIN Dashboard, 2022). This study on the vaccine campaign in Madhya Pradesh (MP), India, aims to capture the key activities, stakeholders, processes, challenges, and learnings to devise a robust vaccination campaign. Government of Madhya Pradesh (GoMP), Clinton Health Access Initiative, Inc. (CHAI), and the Indian Institute of Technology Delhi (IITD) have collaborated to conduct this study on the COVID-19 vaccination campaign in Madhya Pradesh. The system dynamics (SD) modeling approach, which is used to simulate complex systems through feedback loop models and helps devise strategy and policies during adaption to change, was used to conduct an end-to-end analysis of MP’s COVID-19 vaccination campaign.

**Fig. 1:**
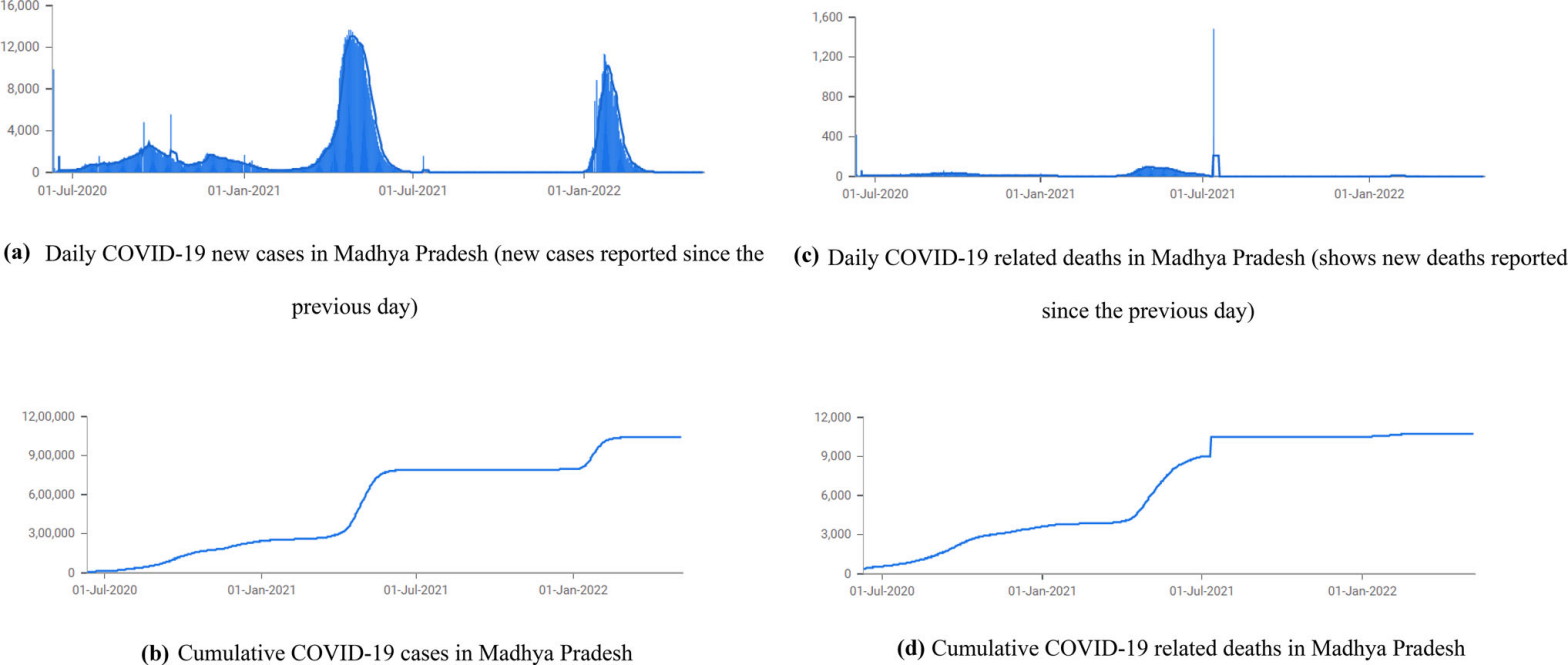
**a**. Daily COVID-19 new cases in Madhya Pradesh (new cases reported since the previous day), **b**. Cumulative COVID-19 cases in Madhya Pradesh, **c**. Daily COVID-19 related deaths in Madhya Pradesh (shows new deaths reported since the previous day), **d**. Cumulative COVID-19 related deaths in Madhya Pradesh

MP is the second-largest state in India by area and the fifth-largest by population and has a diverse rural–urban population with around one-fourth of its area under forest cover. As of March 10, 2022, the total cumulative number of active COVID-19 cases reported in MP is more than 1 million and 10,735 deaths (Fig. 1a–d).

**Fig. 2:**
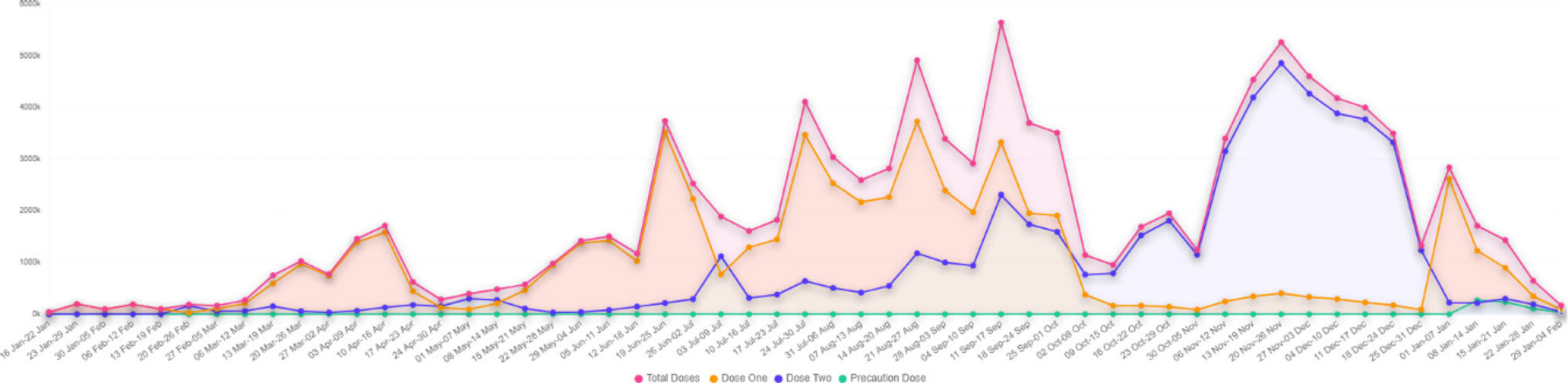
Vaccination doses administered in MP from January 2021 to January 2022 (Source: https://dashboard.cowin.gov.in/)

As of May 5, 2022 (Fig. 2), MP has administered more than 118 million vaccines; from this, 60.2 million were first doses, 56.6 million were second doses, and the remaining were precaution doses.

This campaign involved support from healthcare professionals, frontline COVID-19 workers, and, most importantly, the *Janata* (public) of MP (Mishra, 2021). The participation of the people (*Jan Bhagidaari*) in the state was the central theme of the campaign and was followed at all levels; this called for the support and involvement of frontline and health workers, religious leaders, businesses, social workers, prominent personalities, public, teachers, “*patwaris*” (local record keepers), and self-help groups.

Public campaigns were organized to generate awareness, dispel misinformation, and motivate people to get vaccinated. The GoMP launched multiple COVID-19 vaccination *MahaAbhiyan* (mega vaccination campaign) to mobilize and vaccinate a large number of the eligible population. Wide-ranging strategies were adopted to address local challenges in a decentralized manner to motivate vaccine-hesitant families and increase awareness of the benefits of vaccination (Klafke et al., 2021; Mokline & Ben Abdallah, 2022; Singh et al., 2021; Tam et al., 2021).

This article is laid out as follows. The next section presents a literature review followed by the method and steps followed to build the model. Subsequent sections are the analysis of sub-causal loop diagrams, followed by a detailed qualitative review of the challenges and innovations of the vaccination campaign. The concluding sections include the study’s limitations, the scope of future research, the implications of the findings, and the conclusion.

### Literature Review

COVID-19 vaccination campaigns are a complex process constituting various stakeholders and factors that can impact each other and determine the effectiveness of the end-to-end service delivery. Subsequently, analyzing linkages and feedback loops can help strategize future vaccination campaigns, efficient and effective end-to-end supply chains, service delivery, and demand generation.

The system dynamics modeling approach has been widely used in the healthcare sector, and a literature review conducted between 2000 and 2019 suggests that various healthcare topics have been analyzed using a system dynamics approach (Davahli et al., 2020) and have demonstrated that the complexity of public health can be effectively addressed using this approach (Homer and Hirsch, 2006). For instance, system dynamics simulations have been conducted to provide insights into long-term COVID-19 pandemic outcomes in Malaysia (Salman et al., 2021). A study on the impact of policy innovation implications on health systems during early COVID-19 in Indonesia was conducted using a system dynamics simulation (Aminullah & Erman, 2021), and a qualitative study was conducted on the COVID-19 vaccination campaign in Kerala, India (Elias, 2021). However, there has not been much research using qualitative system dynamics modeling for COVID-19 vaccination campaigns in India.

As a diverse geography country with different climates and demography, India has faced varying challenges during the COVID-19 vaccination campaigns in different states. Different factors such as federal government policies, decisions by state governments, and interventions launched by states and other stakeholders have impacted the vaccination campaigns’ success rates. Therefore, more state-specific studies are needed to understand the vaccination campaigns, and this study aims to understand the COVID-19 vaccination campaign in the state of MP.

## Methods

System dynamics (SD) modeling was developed in the 1950s at MIT by Jay Forrester to simulate compound structures, experiment with models, and devise policies related to adaptation to change (Forrester, 1961). SD modeling is a powerful tool for comprehending and leveraging the interrelation of complex structures through a feedback mechanism. Different scenarios are built to understand possible future outcomes based on past and present situations supported by the assumptions (Sterman, 2000). SD modeling is a complex non-linear relationship of factors that captures information and data flow among various factors. It formulates problems to understand the effect of factors on each other through causal loop linking (Sushil, 1997) and captures qualitative and quantitative details to substantiate the linkages and outcomes. In this study, causal-loop diagrams (CLDs) were developed using a qualitative SD modeling approach to analyze the role of stakeholders and bottlenecks in vaccination campaigns.

### Causal-Loop Diagram

A CLD helps to visualize cause-and-effect relationships among different system components. Variables are depicted as a node, and linkages are depicted through directional arrows. The linkages illustrate how the variation in one variable brings a change in other linked variables. The relationship between two variables can either be in the same direction (marked with a “ + ” sign) or in the opposite direction (marked with a “−” sign). In addition, linkages established from a variable to previous variables in the process flow constitute the feedback loops. These feedback loops are alternatively called causal loops, which can be either “reinforcing loops” (building an overall positive impact) or “balancing loops” (building an overall negative impact).

### Data Sources, Data Collection, and Steps Followed

**Fig. 3:**
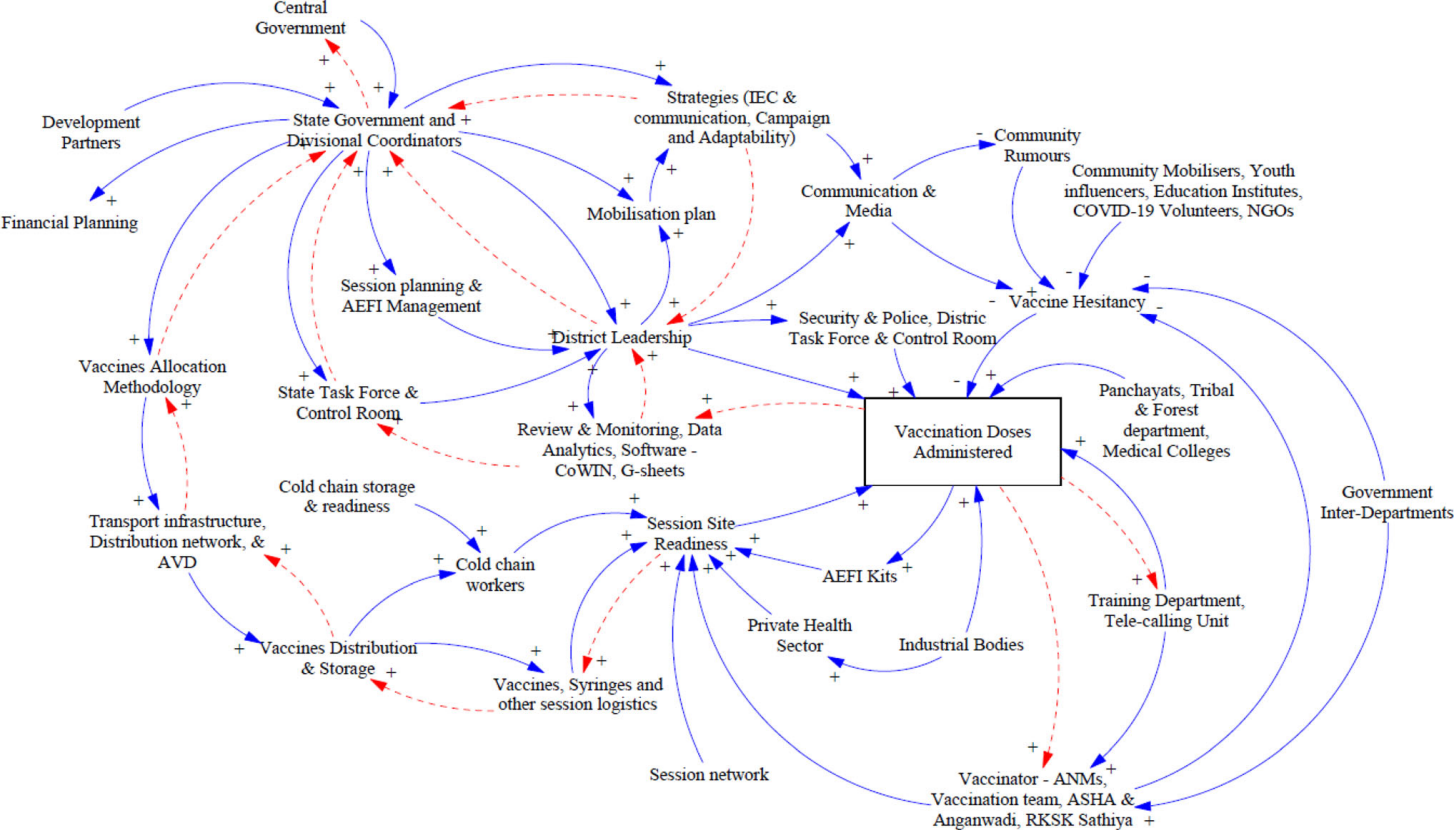
Stakeholders and linkages for the overall vaccination campaign

To gather information for the study, the research team identified data sources, stakeholders, and measures of the vaccination campaign in MP. The primary data were collected through group and individual interviews of stakeholders such as government officials, vaccination teams, immunization supply-chain officials, community mobilizers, volunteers, and development partners. The data collection included all qualitative aspects of the COVID-19 vaccination campaign, and approximately 47 stakeholders were identified and were categorized under four major groups according to their predominant role/responsibilities: policy, infrastructure, communication and information, and monitoring and management levels (Fig. 3). Secondary data were collected included research articles, newspapers, published information, reports, government press releases, and other relevant documents and data. Interviews were recorded (audio) only after receiving consent of the respondent. The interviews were conducted in English, Hindi, and mix of both the languages.

Subsequently, an overall CLD was developed to understand the interdependency of these stakeholders, and sub-CLDs were constructed for the four groups to develop an in-depth understanding based on the primary and secondary data. The main intent of all CLDs is to understand the information flow, policies, instructions, and mobilization plans of these stakeholders^1^ (variables) with an overall impact on the outcome variable “vaccination doses administered.” Semi-structured interviews and group discussions based on open-ended questions were conducted with different stakeholders about the processes and ecosystem. Due to the COVID-19 pandemic, most of the interviews were conducted virtually, and the responses of the interviewees were recorded with the consent of the stakeholders and then individually analyzed and collated by the researchers. In the subsequent CLDs, the blue arrow predominantly represents the flow direction of information or decisions, and the red arrow predominantly represents feedback or reporting. Vensim PLE 8.2.0 software was used to illustrate all CLDs.

### Overall Causal Loop Vaccination Model

Figure 3 depicts the overall CLD, and the main intent of this CLD is to understand the overall linkages of all the identified key stakeholders in the COVID-19 vaccination campaign and the impact of all such variables on the outcome variable “vaccination doses administered.” There are around 149 reinforcing loops, including “vaccination doses administered,” with a minimum of two stakeholders, and some of these linkages are explained below.

The “central government” provided direction and guidelines to the states regarding their COVID-19 vaccination campaign. For instance, extensive guideline documents were released, which provided an overall structure for authorities, roles, and responsibilities for all the constituents and stakeholders. At the same time, states and districts were also advised to customize operational aspects of the guidelines according to their unique infrastructural capacities and local contexts. Similarly, vaccine allocation to the states was decided by the “central government” based on several factors such as infection rate, wastage rate, utilization rate, and eligible population. Once the states received the allocated vaccines, they devised their own “vaccination allocation method” to distribute the vaccines to districts based on their campaign strategy to vaccinate the eligible population as quickly as possible.

The “state government” was responsible for implementing the vaccination campaign and carried out various tasks such as formulating state-specific strategies, devising “vaccine allocation methodology,” building infrastructure, training, planning communication, and cold chain management. “Transport infrastructure,” “cold chain distribution,” and “alternate vaccine delivery (AVD)” networks available for routine immunization were deployed to cater to the needs of the COVID-19 vaccination campaigns. A comprehensive preparedness assessment was conducted to ensure readiness to run routine immunization and COVID-19 vaccination in parallel. For instance, cold storage capacity and additional equipment requirements were estimated based on routine immunization and potential requirements for COVID-19 vaccination.

The presence of highly capacitated and well-funded “development partners” supported the vaccination campaign. For instance, the GoMP, in collaboration with development partners, formulated an operational plan considering factors associated with vaccines and cold chain distribution, human resources planning, delivery strategy, and infrastructure planning that created an overall positive impact on the vaccination campaign. “Cold chain workers” at various levels supported various critical aspects of “session site readiness,” including managing vaccine transportation, cold storage of vaccines, managing return of unused inventory, and data entry on the electronic Vaccine Intelligence Network (eVIN)^2^ portal. The “State government” issued guidelines on “session planning” and “adverse effect following immunization (AEFI) management,” which are then communicated to district leadership and further to sub-district level. The “state task force” and “control rooms” supervised the divisions, districts, and blocks through real-time review and monitoring of “data analytics fetched from CoWIN App” to facilitate the tracking of vaccination progress and timely problem resolution.

“State government and “district leadership” developed a comprehensive “mobilization plan, IEC, and communication strategy,” which were crucial for increasing “vaccination doses administered.” “Communication & media” helped curb community rumors about COVID-19 vaccines, which in turn reduced “vaccine hesitancy” and had an overall positive influence on the vaccination coverage. For instance, various innovative media awareness programs helped people understand the importance of vaccinations, especially in rural areas of MP.

“Security & police” helped with crowd management and monitoring sessions while “district task force & control room” supported campaign planning and management, handling grievances, and reporting to the authorities. “Vaccinators-Auxiliary Nurse Midwives (ANMs),” “vaccination teams,” “Accredited Social Health Activists (ASHAs) and *Anganwadi* workers (AWWs),” and “*Rashtriya Kishor Swasthya Karyakram* (RKSK; National Adolescent Health Programme) *Sathiya*” all supported in ensuring “session site readiness” and reducing “vaccine hesitancy.” The departments conducted training programs regularly to communicate revised or new guidelines. “Government inter-departmental” collaboration supported frontline workers to reduce vaccine hesitancy and community mobilization through their resources. For instance, “panchayats” and “tribal & forest department officials” helped “community mobilizers” to bring awareness and coordinate with populations living in tribal and forest areas.

## Sub-Causal Loops Diagram Analysis

Different sub-sections in this section analyze each sub-CLD and the influence of stakeholders in the vaccination campaign. Semi-structured interviews with various stakeholders were used for the data collection.

### Data Collection

The basic theme of the conversations revolved around stakeholders’ roles, challenges, solutions, innovations, vaccine hesitancy, community mobilization, infrastructure, future challenges, and suggestions for improvement. Table 1 lists all the stakeholders interviewed for this study; thirty-four interviews were conducted, and the duration of the interviews was between 20 to 60 min.

**Table 1:** List of stakeholders who were interviewed

No	Sub-CLD Group*	Stakeholder	Role
1	Policy (Planning and implementation)	State government	Stakeholder
2		Divisional Coordinators	Stakeholder
3		Development partners	Stakeholder
4		District leadership	Stakeholder
5		Control rooms & Taskforces	Stakeholder
6	Infrastructure (Physical, HR, and Technological)	Vaccinator—ANMs	Stakeholder
7		Vaccination team	Stakeholder
8		Cold chain workers	Stakeholder
9		ASHA & Anganwadi	Stakeholder
10		NGOs	Stakeholder
11	Communication and Information	RKSK Sathiya	Stakeholder
12		Community Mobilizers	Stakeholder
13		Panchayats	Stakeholder
14		Communication and Media	Stakeholder
15		COVID Volunteers	Stakeholder
16	Monitoring and Management	Training Department**	Stakeholder
17		Tele-calling unit	Stakeholder

*Stakeholders were categorized into four groups according to their predominant role/responsibilities; however, they may have additional roles in more than one sub-CLD group. **Training Department is not a single entity and comprises all the departments or institutes at national/state/district/block levels that provide human resources training to the private health sector, NGOs, and volunteers

Interviews were based on open-ended questions (Online Appendix 1, Table A1), and follow-up questions specific to the roles and responsibilities of the stakeholders evolved during the conversation. The following sub-sections demonstrate the CLDs resulting from the secondary data research, discussions within teams, and interviews with stakeholders involved in the vaccination campaign. The main intent of each sub-CLD is to understand the impact flow from all respective variables on the outcome variable “vaccination doses administered” (Online Appendix 2, Table A2).

### Sub-CLD for Policy Group

**Fig. 4:**
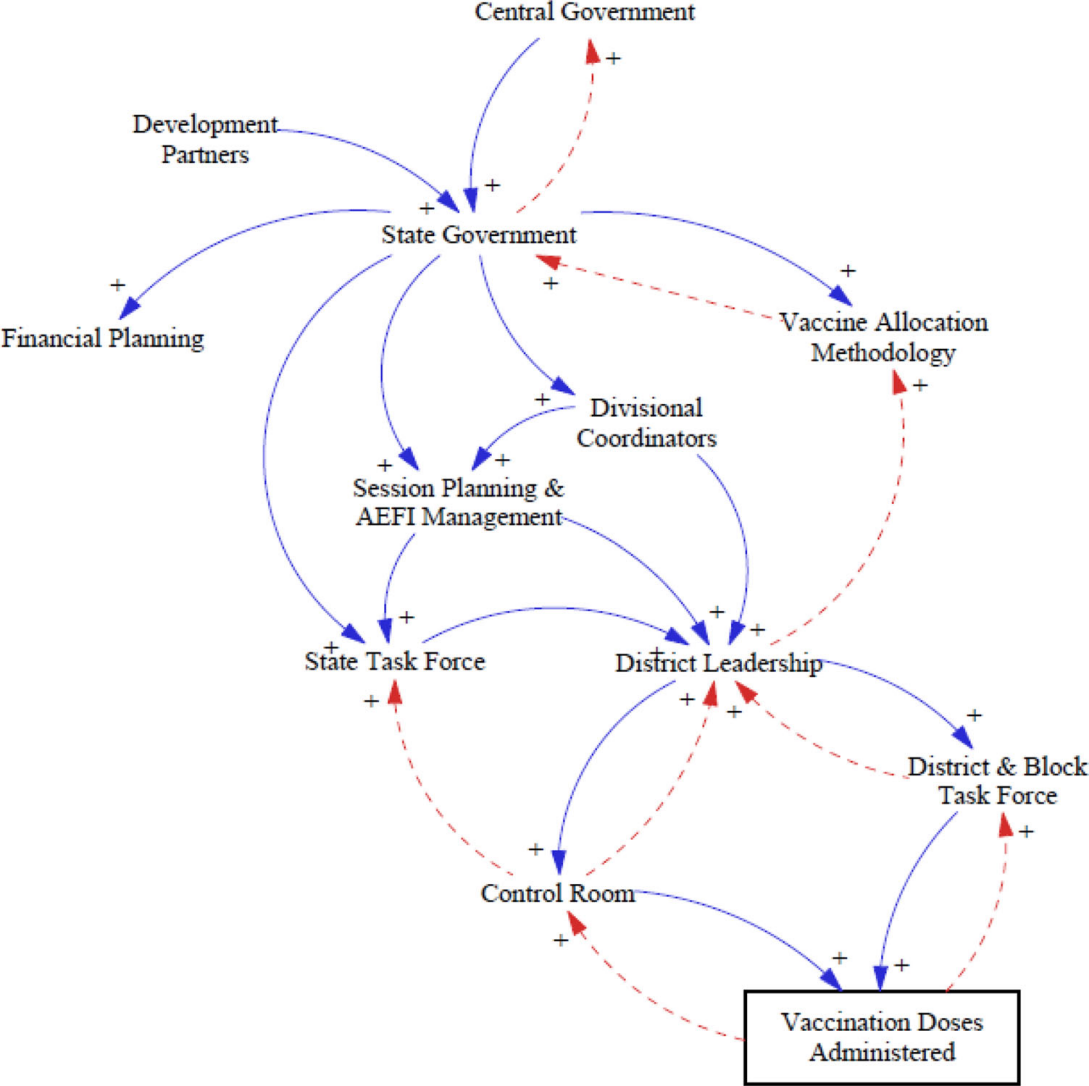
Stakeholders and linkages for policy groups

Figure 4 illustrates how different policy level stakeholders are linked. As illustrated by this sub-CLD, policy decisions taken by the “Central Government” to improve vaccination coverage directly influence the implementation decisions taken by the “State Government.” A positive linkage between “Development Partners” and “State Government” denotes how the Development Partners’ experience and technical expertize helped in conducting evidence-based planning, timely preparation, and optimizing limited resources for the state:

Development partners such as CARE, CHAI, JSI, KPMG, UNDP, UNICEF, and WHO supported to frame vaccination plan, infrastructure preparedness assessment, and data analytics...

Similarly, actions and decisions of the “state government” directly influence the subsequent responsibilities of the “district leadership.”

For example, “Political will” played an important role in successfully implementing the COVID-19 vaccination program in MP, where the administration remained agile, conducted strict monitoring, and took quick data-backed decisions based on the evolving situation. One of the state-level respondents stated: “The Honorable Chief Minister’s (CM) political will get translated to the administration will…follow-up and review on a daily basis were done by the CM with district leadership and in the cabinet too…” Likewise, the “Vaccine Allocation Methodology for districts is dynamic and encompasses several key variables, such as “prone to high-risk populations,” “vaccine consumption capacity,” “health infrastructure,” “existing vaccine stock sufficiency,” “vaccination coverage targets,” and “local planning and campaigns”: “Eight to ten parameters, such as COVID-19 cases, vaccine consumption run rate, performance factors, were identified to allocate vaccines….”

“State government” and “district leadership” collectively decided the session planning for the vaccinations based on the district-wise demand-supply metrics. “Session planning” involved planning the number of sessions on any given day, the location of such sessions, availability of human resources and other resources, and vaccination coverage targets. Moreover, routine immunization (RI) activities were also managed and not disrupted during the pandemic. For instance, the two RI days in a week were earmarked for RI efforts, and only minimal COVID-19 vaccination work was conducted on those days: “The entire team has worked hard continuously for more than six months, and efforts have been made to conduct COVID-19 vaccinations along with RI… frontline workers have even worked on Sundays…”

Like the Central-State government relationship described above, the policy decisions taken by the “State Government” directly influence the responsibilities of the “Divisional Coordinators,” who manage the day-to-day functioning of the “Control Rooms,” all of which ultimately led to improved numbers of “vaccination doses administered.” Similarly, one of the roles of district and block “Control Rooms” were to resolve grievances, answer queries, and provide necessary information to people related to the vaccination campaign. “Control rooms” solved such queries because of effective communication about policies, guidelines, and processes from the “State control room and Divisional Coordinators.”

“Control Rooms” and “District and Block Task Forces” reported their learnings and unresolved queries to the “State Control rooms” and “Divisional Coordinators,” who could then use the ground-level information to suggest relevant guideline revisions and innovative ideas to improve vaccination coverage, thus forming the “feedback loop.” The “vaccination doses administered” also formed a part of this feedback loop, as this information was available on CoWIN, and key metrics like coverage and utilization were analyzed to make data-driven policy decisions at the state level, all of which culminated in an overall improvement in vaccination coverage. One respondent said: “Vaccine consumption was tracked on a daily basis by control room teams and feedback was given to district and state leadership… brainstorming was done to maximize vaccination coverage and flexibility was given to the district leadership…”

### Sub-CLD for Infrastructure Group

**Fig. 5:**
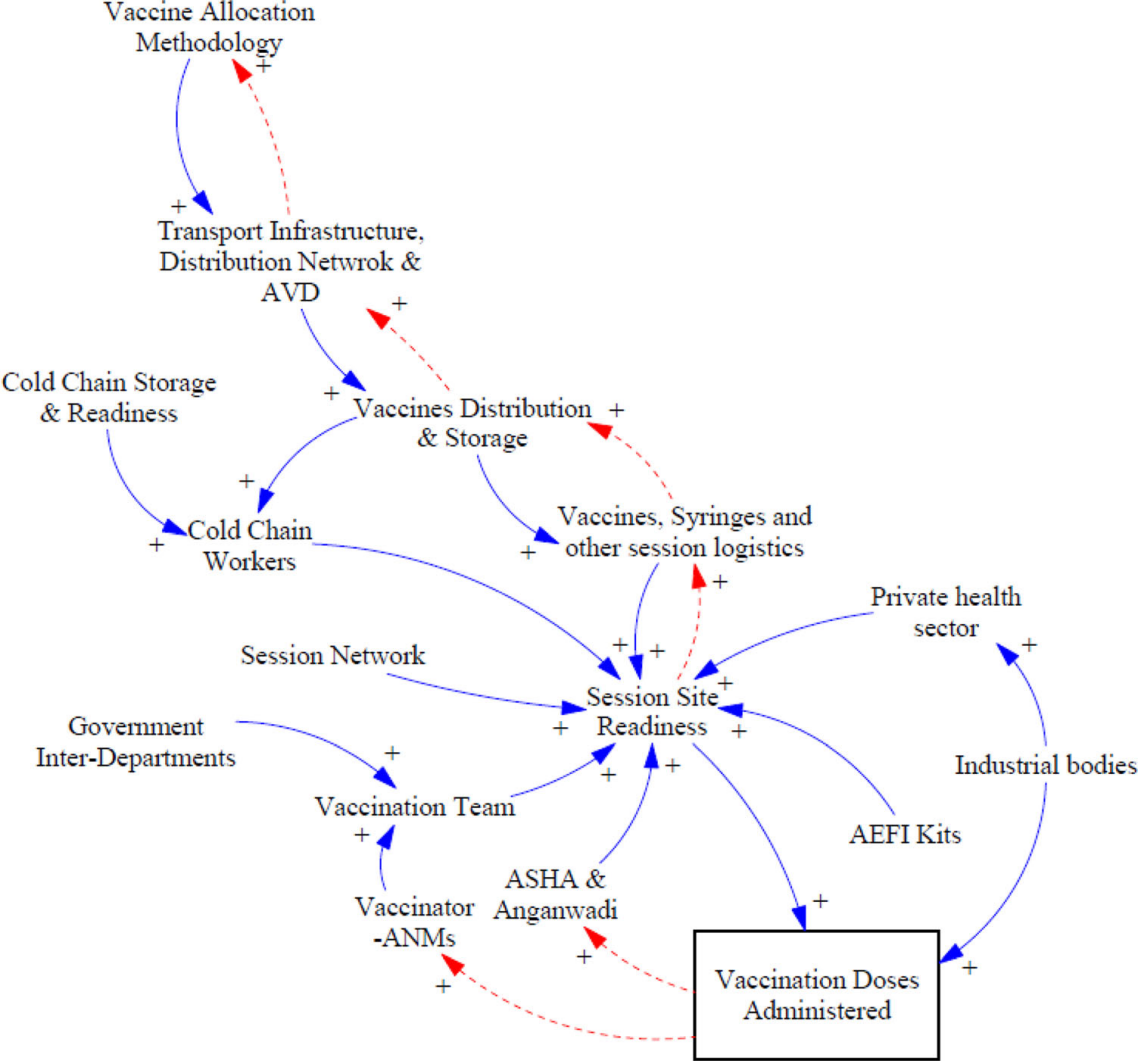
Stakeholders and linkages for the infrastructure group

Figure 5 illustrates how different infrastructure level stakeholders are linked, and this group includes physical infrastructure and human and technological resources.

Ensuring *“*cold chain storage and readiness” was important for the smooth functioning of the campaign as this directly influences the overall vaccine logistics available at the ground level. Once vaccines were distributed to the districts and session planning, the “cold chain workers” handled the end-to-end supply of “vaccines, syringes, and other session logistics” to the session sites and helped develop “session site readiness.” One state-level respondent also appreciated the work of vaccine van drivers: “Drivers of vaccine vans have also worked continuously 24/7 to deliver vaccines on time….”

The preparation of micro plans is important so that the session sites can be published on CoWIN, which gives time for mobilizers to generate demand and cold chain handlers to manage vaccine supply and session site readiness, ultimately leading to a positive effect on the overall “vaccination doses administered.” Throughout the campaign, strict protocols around updating vaccine stock and temperature control monitoring on the eVIN portal were followed, which helped ensure safe storage, transport, and vaccine distribution as per the guidelines. Backup plans were always in place to address unforeseen circumstances, and a respondent told us: “Frontline workers were instructed to shift vaccines in cold boxes or to nearby storage sites during a long power failure in certain rural areas so that temperature control for vaccine stocks can be ensured….”

Cold chain handlers, cold chain technicians, and vaccine cold chain managers ensured that vaccine stock planning, cold chain equipment maintenance, and vaccine distribution were smoothly managed. They also ensured that vaccine boxes were clean, not decolorized, included proper coding on the vaccine boxes, and took utmost care while returning unused vaccine vials. One respondent said: “The vaccine distribution and logistics process is highly sensitive and a key aspect of the whole vaccination process… huge risk is associated with it…” The unused “vaccines, syringes, and other session logistics,” “AEFI Kits,” and other disposable items are sent back through the reverse supply chain from session sites to the cold storage, which forms the main feedback loop of the CLD. This feedback loop shares new ideas and challenges with the “state government” that assists with policy decisions. For example, one respondent said:

Initially, certain vials of Covishield had an extra eleventh dose in a vial. The vaccination team was unsure how to report such incidents, and additional extra vaccines were wasted… when this was brought in cognizance of higher authorities, guidelines were issued that the eleventh dose can be used, recorded, and reported…

Under the advisory of the Government of India, MP also minimized vaccine wastage. “ASHAs” and “AWWs” were guided to mobilize people so that sufficient beneficiaries were present in one session slot^3^: “ANMs and vaccination team were suggested by cold chain officials to open vials once a sufficient number of beneficiaries were present on-site…” In rural and tribal areas, “ASHAs,” “AWWs,” “ANMs,” “vaccination teams,” and “government inter-department collaboration” such as the forest department, education department, and revenue department supported the vaccination program to increase awareness among the public. For instance, “*Khatla Baithaks*” (*Charpai *or ‘cot’ meetings) were held in tribal areas, which helped communicate the benefits of getting vaccinated. Inter-department coordination within the government has been one of the most important pillars of MP’s COVID-19 vaccination journey. As one respondent said:

During the lockdown, most of the government departments, administrative offices, revenue departments, municipal panchayats, and education departments weren’t fully functional… thus, the human resource of these departments was deployed in the vaccination process that helped to improve the vaccination coverage… now when there is no lockdown, and these departments are fully functional then such support is diluted…

The private health sector has a strong network across many cities; therefore, existing private health facilities were leveraged as COVID-19 vaccination centers (CVCs) to maximize the effectiveness of the campaign. One respondent stated: “We contacted and converted private health facilities into CVCs… for instance, in Bhopal there are more than 350 private nursing homes… they have networks, expertize, public relation, and resources….”

### Sub-CLD for Communication and Information Group

**Fig. 6:**
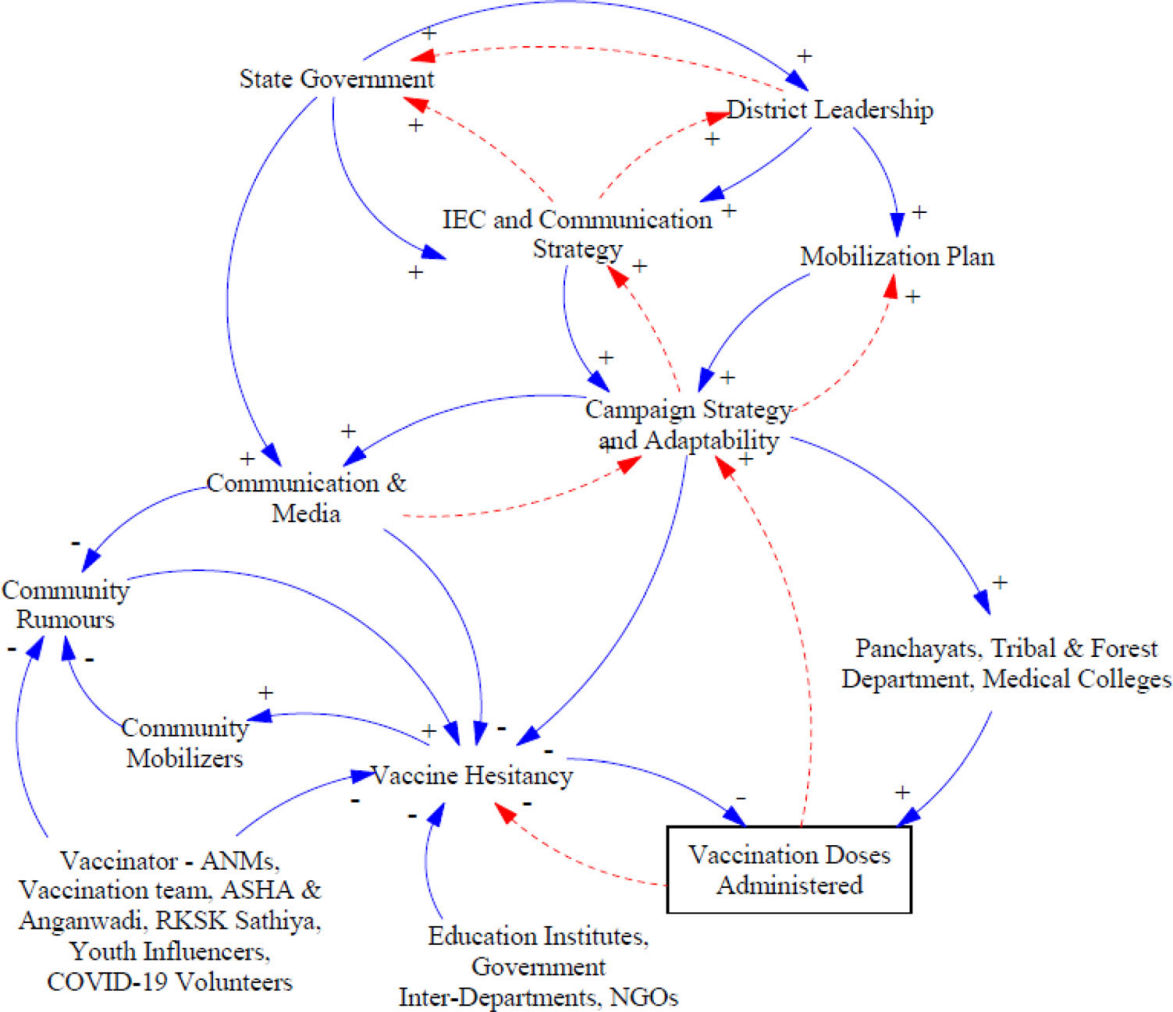
Stakeholders and linkages for the communication and information group

Figure 6 illustrates how different communication and information level stakeholders are linked. This sub-CLD includes “state government” and “district leadership” stakeholders predominantly part of policy level sub-CLDs. Most of the guidelines toward “mobilization plans” and “IEC and communication strategy” were framed centrally by the “state government.” However, following the “campaign strategy and adaptability” aspect, districts were advised to adopt communication and mobilization plans according to region-specific challenges. One respondent said:

We organized ‘*nukkad nataks*’ (street shows) in rural areas… helped people during the lockdown crisis… supported the administration in every possible capacity…

Additionally, “COVID-19 vaccination *MahaAbhiyans*” were established as a key strategy of the State’s “mobilization plan.” A MahaAbhiyan is a mega campaign day when the vaccination coverage targets are much higher than routine days, and the entire administration becomes involved with support from various government departments beyond the health department. The GoMP reported a record number of vaccination doses administered during these mega vaccination campaigns. Following the success of the state-level campaigns, districts also started organizing their own localized campaigns in consultation with the “district leadership” to boost their vaccination pace: “We suggested that the leadership organize a ‘*Zila*’ level *Maha Abhiyan* to increase vaccination coverage….”

Based on the primary research, it was found that initially, the public was hesitant about vaccinations or unaware of the campaign, especially in forest and rural areas. Almost every frontline worker witnessed vaccine hesitancy during their field visits: “The rural and tribal population was unaware of the COVID-19 disease and vaccination and thus were reluctant and hesitant toward the vaccines… many of them believed the vaccine can even cause death… they needed assurance for their health safety post-vaccination….”

The “IEC and communication strategy” has helped to address “community rumors” and “vaccine hesitancy.” The on-ground workforce, such as “vaccinator-ANMs,” “vaccination teams,” “ASHAs,” “AWWs,” “RKSK Sathiya,” “youth influencers,” and “COVID-19 volunteers,” have helped to reduce “vaccine hesitancy” and prevent “community rumors,”^4^ therefore demonstrating a negative relationship. Local traditions, customs, and rituals were followed to engage people and effectively communicate vaccination benefits. The following respondent comments confirmed this: “Announcements through religious places, municipal vehicles… at one instance one respondent stopped a marriage procession to announce and request people to follow COVID-19 appropriate behavior…”; “Following a local custom, ‘peele chaawal’ (yellow rice) were given to villagers as an invitation to get vaccinated at session sites. Villagers who accepted the invitation were self-motivated to get vaccinated…”; “Village heads and family members were vaccinated in front of other villagers…”; and “Repeated continuous visits to motivate people….”

“Education institutes,” “government inter-departments,” and “NGOs” have a negative relationship with “vaccine hesitancy” since their increased involvement leads to reduced vaccine hesitancy. Such organizations coordinated with village locals to arrange awareness camps, meetings, and group discussions. “Panchayats, tribal and forest departments” have helped in preventing rumors and bringing awareness by leveraging their staff and infrastructure. The respondents also acknowledged the support from other departments: “The infrastructure of the AWW offices and panchayats were utilized as vaccination centers and for mass meetings to bring awareness…”; “During a visit in a forest area… help from a forest official was sought as people listen to them and consider them as police… often used wireless trans-receiver to contact team members…”; and “We used to discuss vaccination visit-related problems with panchayats… they used to listen to our problems and support us…”

Almost every respondent appreciated the support of the public and administration: “Support from the authorities, seniors, village locals, team members, and volunteers, helped to motivate the public to get vaccinated… it is a collective effort…” The role of “communication and media” has also been observed to prevent “community rumors” and reduce” vaccine hesitancy”; for instance, awareness campaigns on national television, radio channels, and local newspapers helped build trust among the public in rural areas: “We used online medium, mobile, WhatsApp, to bring awareness among the public… that resulted in reduced vaccine hesitancy….”

In forest areas and difficult terrains, since there is a network issue, the only medium available was to personally communicate and educate people about vaccination, and in such areas, Panchayat officials, forest department officials, and local NGOs played an important role in generating awareness: “I searched for NGOs from a list in newspaper and contacted them… we worked together to bring awareness and motivate people for vaccination…” and “We counseled children and students about the importance of vaccination… educating and sharing of benefits of vaccination reached through student groups to their parents….”

The “campaign strategy” always remained agile and adapted according to the ground situation. For instance, when the public did not turn up for the second dose, it was found that they did not want to stand in long queues along with first dose beneficiaries, which were more in number. Then, it was decided that second dose beneficiaries would either have separate queues and would be given priority with no waiting time. The work of frontline workers has been acknowledged by all respondents. Field staff supported vaccinations, mobilization, addressing vaccine hesitancy, managing crowds, and many other efforts. The constant motivation of healthcare and frontline workers who are responsible for all implementation is a key aspect of a successful campaign: “ANMs, ASHAs, AWWs, vaccination teams are working hard despite various hardships… they make numerous field visits… manage crowds at vaccination centers… vaccinate so many people that they become exhausted… we appreciate their hard work…” and “Difficult terrain and network issue in rural and forest areas led to increased working hours… safety for female staff was a major issue in such areas and during extreme weather condition such as overflowing of rivers.”

Additionally, the utility of a token system was observed at the vaccination sites. People were called for vaccination according to a token number rather than waiting in a long queue, as mentioned by many respondents: “Tokens were distributed in rural areas by ANMs and AWWs either a day before or in the early morning on the day of the scheduled vaccination… this has helped to improve in vaccination coverage and minimized vaccine wastage due to low turn-up of beneficiaries….”

### Sub-CLD for Monitoring and Management Group

**Fig. 7:**
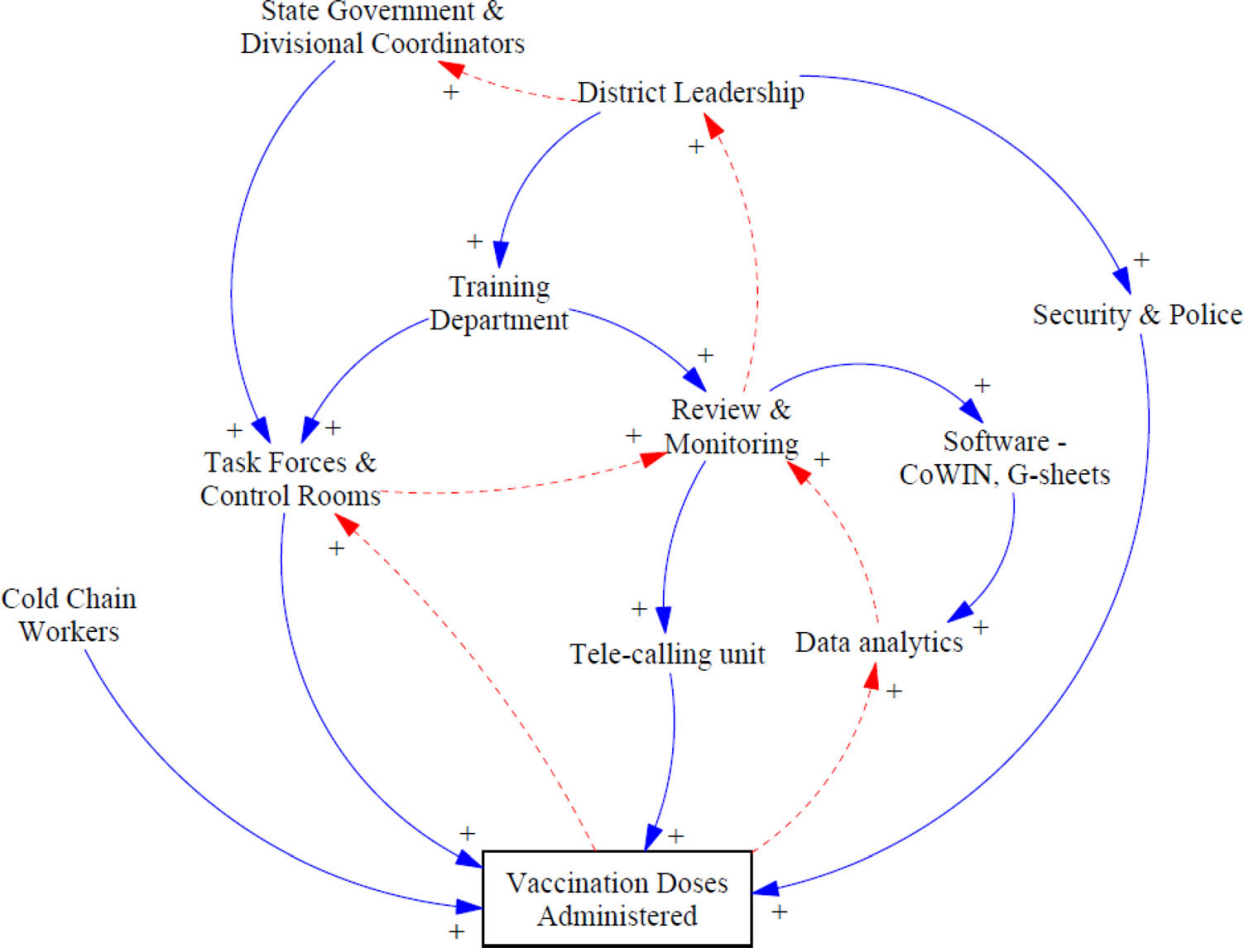
Stakeholders and linkages for monitoring and management groups

Figure 7 illustrates how different monitoring and management level stakeholders were linked. Training programs were organized for state/district/block level human resources across different departments, including the private sector, NGOs, volunteers, and others. Timely training was conducted on software used extensively in the campaign like CoWIN and eVIN, which supported efficient data entry and vaccination coverage data reporting.

Due to the pandemic, many virtual training sessions were conducted to improve understanding and communication. For example, one of the data entry operators said: “Training was given to us regarding the CoWIN app, and since the app has also improved a lot, now it is easy to handle query…” Another respondent said, “During the training, he was the only participant from a rural area and was the first one to form demo sessions in the CoWIN app among all the participants… he was appreciated by the instructor for quickly and correctly grasping the technology… he felt motivated by such recognition…” The respondents from the cold chain supply chain said: “Regular and frequent communication was maintained with the vaccination team and AVDs to keep track of the stock and data of the vaccines and other items…” Task forces, control rooms, and tele-calling units at the state and district levels were also orientated and kept updated using revised guidelines: “Issues raised by the callers were adequately answered by concerned persons… if they require, then help from other departments was also sought….”

Initially, usage time post opening of vaccine vials was six hours, but this was changed to four hours. Such revised guidelines were communicated to the frontline workers, and they were trained accordingly. The impact of this training directly influenced the number of “vaccination doses administered.” Another state-level respondent said: “Cold chain technicians were trained by National Health Mission^5^ officials, additional tools and budget were provided to improve efficiency… daily monitoring and review of equipment downtime….”

Vaccination data through the CoWIN portal were reviewed, monitored, and analyzed daily, and insights from the available data became the foundation for a stringent “review and monitoring system” at the district and state levels. Daily morning reports, hourly reports on campaign days, weekly performance reports for district review meetings, composite index-based performance analyses, and many other such “data analytics” methods/tools were used by the “state government” and “divisional coordinators” to convey data-backed insights to the “district leadership.” Data from the CoWIN and other reporting tools such as Google sheets were used and analyzed extensively to generate insights related to session planning, HR capacity utilization, second dose due trends, and wastage rates.

More data operators were required, especially during the COVID-19 vaccination “*MahaAbhiyans*,” and “district leadership” addressed this challenge by appointing additional data entry operators. The “telecalling unit” contacted eligible beneficiaries, counseled beneficiaries, resolved queries, and gave them information about the vaccination sessions and schedules. Relevant information like the location of the session sites and timing were provided, and other queries were handled up-front to save time, especially on daily wagers. One respondent highlighted: “Daily wagers leave home early in search of work so typically they have one-two morning hours to get vaccinated… we arranged early hours on-site sessions for them….”

Volunteers were engaged to lend support to the telecalling units. The self-motivation and importance of volunteers can be understood by a statement made by one of the respondents: “We have served with all our hearts… the whole team has worked together… we called extra people during Maha Abhiyan… they still ask… when do we have to come again… just let us know, and we will be happy to come…” “Security & police” officials were deployed at session sites for crowd management. For instance, during the second wave of COVID-19 (Apr–May 2021), high demand was observed at many session sites, which created chaos. People wanted to get vaccinated as soon as possible, but the vaccine supply was limited. “Security & police” officials helped in crowd management so that the vaccination teams could focus on their work.

## Discussion

In the early weeks, the vaccination pace slowed due to the limited supply of vaccines, vaccine hesitancy, and limited understanding of the guidelines. Based on discussions with stakeholders, some potential reasons behind vaccine hesitancy identified were community rumors, myths behind vaccine efficacy, poor awareness around the benefits of vaccination, and fear of catching COVID-19 infection when exposed to crowds at places like hospitals or COVID-19 vaccination centers. Efforts by all stakeholders, improved supply, and dynamic program modifications gradually led to improved COVID-19 vaccination coverage in MP. An in-depth analysis of the challenges, innovations, and best practices has provided a holistic picture of MP’s COVID-19 vaccination campaign.

### Challenges, Innovations, and Best Practices

MP achieved a COVID-19 vaccination coverage rate of more than 90% of the adult population in 12 months. Vaccine hesitancy was addressed through awareness generation and innovative practices.

#### Central Government

The directions, operational guidelines, and standard operating procedures (SOPs) released by the Government of India set the foundation for the COVID-19 vaccination campaign, which was adopted by all states in India. In addition, the COVID-19 vaccination campaign saw a first-of-its-kind information system through the CoWIN app, which was used extensively throughout the vaccination campaign and helped with data management and analytics. Similarly, the eVIN portal played a key role in efficiently managing vaccine stock across the country. Such advanced systems enabled data analyses at the district and state levels and promoted data-based decision-making.

#### State Government, Divisional Coordinators, and District Leadership

**“**State government and district leadership” ensured the creation of adequate vaccination sites, vaccination teams, sufficient vaccine delivery vans, AVDs, security, and volunteers, which helped to ensure the readiness of the infrastructure before the launch. During the pre-vaccination preparations, the GoMP ensured the availability of sufficient equipment at all cold chain points to account for contingencies. In addition, providing 10 L of petrol per month to cold chain technicians at the district level made it easier to repair on-site defective cold storage equipment. Further, initiatives were taken by the GoMP to provide lunch and dinner facilities for staff.

Challenges related to vaccine hesitancy, such as low demand and insufficient community mobilization, were addressed by the GoMP through various initiatives and innovations. For instance, drive-in vaccination facilities helped people with special needs and older people, and inter-personal communication helped reduce vaccine hesitancy among the diverse population of MP. Targeted strategies based on geographic and demographic factors (like urban, rural, tribal, or forest populations, and gender or age groups) were developed to reduce vaccine hesitancy. Other challenges related to logistics and high vaccine wastage were also addressed. Similarly, the timely dissemination of new information, revised guidelines, and CoWIN app updates were critical to ensuring smooth and consistent communication at all levels. The GoMP solved this by conducting trainings in hybrid (physical and virtual both) mode and undertaking live online demonstrations to mass end-users (verifiers and data managers) related to CoWIN app updates or other portals. Challenges related to human resource burnout/fatigue were addressed by involving volunteers and partnerships with NGOs to become a part of the vaccination team as verifiers or data entry operators.

The findings demonstrate some key strategic initiatives for improving COVID-19 vaccination coverage in the state. First, MP adopted the “*Jan Bhagidaari*” (public participation) theme for its COVID-19 vaccination campaign. This involved participation from people throughout the state in getting vaccinated and getting others vaccinated. Under the *Jan Bhagidaari* philosophy, the GoMP instituted “Crisis Management Groups” during the second wave of the COVID-19 pandemic in India (April 2021 to June 2021). These were at Panchayat, block, and district levels and had representation from every level of stakeholders. Their close connection with the public established was leveraged to mobilize beneficiaries during the campaign.

Similarly, the state focused on youthful energy by involving “*Saathiya* volunteers,” who are part of the “*Rashtriya Kishor Swasthya Karyakram*.” “*Saathiya* volunteers” across 13 districts, using innovative means such as street shows and wall-painted slogans, and they made the community aware of the importance of vaccination. The Saathiya volunteers often accompanied health care workers and officials on door-to-door visits, registered beneficiaries at session sites, and aided in the vaccination of senior citizens. Lastly, MP ensured the participation of COVID-19 volunteers through digital tools and local initiatives to raise awareness and promote focused mobilization for the second dose. “*Mai Hu Corona Volunteer*”^6^ (I am Volunteer) campaign was a key innovation toward leveraging volunteers and youths in mobilizing beneficiaries.

Second, COVID-19 vaccination “*MahaAbhiyans*” (mega vaccination campaigns) were organized to provide momentum in vaccination coverage by organizing once-in-a-while mega campaigns to identify clusters of the left-out population, generate mass awareness, ensure wider availability of vaccines, and push the entire state machinery toward this campaign.

Third, localized traditions and rituals like “*Khatla Baithaks*” (*Charpai* or ‘cot’ meetings) were organized in villages to encourage locals through public meetings and discussions with local influencers, village heads, and prominent local personalities. Similarly, “roko toko abhiyan” (stop and remind campaign) was started to stop, remind, and encourage people to get vaccinated.

Fourth, there was a high focus on stringent data monitoring and review at all levels from the start. The state and districts maintained parallel Google spreadsheet reporting from the start, as a backup and for reporting, which helped in data-backed decision-making, thus demonstrating a very early decision to rely on data insights for improved strategy. The composite index ranking method was adopted to accommodate all key performance indicators and other metrics, providing districts with data-backed guidance on their improvement areas and strengths.

#### Development Partners

Various Development Partners^7^ (like CARE, CHAI, JSI, UNDP, UNICEF, WHO, and others) supported the state and district leadership in early preparedness for the efficient and effective execution of the COVID-19 vaccination campaign. Early preparedness focused on calculating funds requirements, infrastructure enhancement planning, human resource capacity assessment, budget planning, and capacity augmentation related to storage and transportation of vaccines and ancillary supply. During the campaign, development partners supported in various aspects like data analytics, software training, communications, frontline workers training, campaign documentation, monitoring, supervision, and stock management. Support from development partners was pervasive and existed not just at the State level but also at the district and sub-district levels in terms of ground support at COVID-19 vaccination centers.

#### Cold Chain Workers (VCCM, CCH, CCT, and DVSK)

Cold storage supply chains require strict due diligence at every vaccine stock maintenance and management level. Regular follow-ups by cold chain workers with on-site teams helped develop efficient vaccine stock management.

#### Vaccinator-ANMs, ASHAs, and AWWs

Mobile vaccination teams deployed in remote areas and building trust among villagers by vaccinating frontline workers’ family members and prominent locals in front of villagers has helped in improved vaccination coverage.

#### Community Mobilizers, Panchayat, NGOs, COVID-19 Volunteers, RKSK Sathiya, and Verifiers

Lists of COVID-19 volunteers and NGOs were published in the media by the GoMP, and Panchayat rooms and offices were utilized for public awareness meetings and session sites. Community mobilizers and NGOs formed a human chain in remote areas to make people aware through word-of-mouth, and volunteers who worked during “*Swachchh Bharat Abhiyan*” were contacted to help in community mobilization. There was respect and trustworthiness among villagers for volunteers and NGOs who helped during the second wave. A slogan such as “I am vaccinated” was publicized, images in print media, and NGO members in an identifiable uniform were other innovations that helped in community mobilization. The extensive use of content on digital platforms, such as short informative videos over WhatsApp, was also used. Field staff shared ground experiences in peer groups/other villages, used WhatsApp status updates to bring awareness, and utilized lockdown time to bring awareness through students/children to their parents. Local customs like “*peele chawal*” were also used to motivate people to get vaccinated.

#### COVID-19 Command Center (CCC) and Tele-Calling Unit

A COVID-19 command center that worked during the outbreak of the COVID-19 pandemic was utilized for tele-calling and addressing grievances related to COVID-19 vaccination. Multiple teams were formed that constituted doctors, teachers, operators, and technical experts who worked 24/7 in shifts, and voters’ lists were used to identify left-out beneficiaries who were called and motivated to get vaccinated.

#### Government Inter-Department Coordination

Police and education departments supported community mobilization, and “tribal and forest departments” supported in contacting team members and volunteers through wireless phones during field visits. All departments motivated their own staff and networks to get vaccinated, and support from crisis management groups was crucial in identifying individuals left out, generating mass awareness, conducting social audits and surveys, mobilizing demand during MahaAbhiyans, and motivating people to take the second dose.

### Foreseen Challenges, Recommendations, and Suggestions

Besides innovations and best practices, understanding future challenges and the scope of improvement are also important. Valuable recommendations and suggestions to foreseen challenges of the campaign were received from the stakeholders and ground staff. Vaccination demand became sluggish after 90% + of the population was vaccinated, and it is difficult to identify the left-out population due to migration, death, target mismatches, and other factors. The festive season in October 2021 also affected the vaccination pace, and therefore, such events should be considered for future vaccination campaign(s). Knowledge and information needs are dynamic and should be provided through appropriate communication platforms, of which inter-personal communication played a major role, as most populations in remote/rural/tribal areas are not digitally literate.

The frontline workers’ worked long, extended hours, seven days a week. This will eventually lead to burnout, and thus adequate incentive and recuperation mechanisms need to be put in place. Traveling to difficult terrain and in extreme weather is risky and unsafe, and hence safe and better transportation facilities along with basic amenities at session sites should be provided. There were several exogenous challenges, such as interruption of transport facilities (rail, bus).

Most of the interviewees acknowledged that higher authority appreciation was encouraging and recognizing the workers for their efforts as it gives a sense of belongingness. In some cases, the GoMP recognized the efforts through appreciation certificates, but identifying each contributor and ensuring appropriate rewards and recognition mechanisms is challenging. This needs to be resolved, and it is extremely important to recognize and reward all the campaign heroes. Data will become a more central role in all public health areas going forward, so upskilling healthcare and frontline workers in terms of digital literacy and ease of usage will be key. Similarly, the accessibility of vaccination certificates in rural and tribal areas, where people cannot operate digital tools to download certificates, will need to be solved.

### Implications

Vaccinations and non-pharmaceutical interventions substantially impact future COVID-19 cases, hospitalization, and deaths (Moghadas et al., 2021; Sandmann & Jit, 2021). Governments can consider these models and linkages and develop strategies to improve vaccination coverage. The challenges identified in this study can help policymakers and administrators provide improved services. Innovations in one region can be implemented as best practices in similar regions. The key identified factors of the COVID-19 vaccination campaign can help devise a model for other immunization programs. The foreseen challenges and suggestions can help the government and administration to prepare for upcoming vaccination campaigns. The CLD models and analysis will help understand the vaccination campaign. The stakeholder innovations can improve policy implementation and innovations at all levels for future vaccination campaigns.

Overall, when the best practices are implemented, the public will benefit from better healthcare management, and governments across the globe can get real insights into the end-to-end modeling of the vaccination campaign in MP, including the main input, output, and impact of sub-CLDs (Online Appendix 3, Table A3). It helps develop an understanding of the relationships among stakeholders, their roles, responsibilities, and accountability in the system.

### Limitations and Scope for Future Research

This study was conducted in MP, a state with a distinct geography and specific challenges, which might differ from other states. CLDs for other states, if modeled separately, will constitute most of these stakeholders; however, a few MP-specific stakeholders might not be applicable. Similarly, local innovations in MP might also differ. Stakeholders have been positioned according to their predominant role in sub-CLD groups but can have an overlapping presence in more than one sub-CLD. In addition, the categorization of stakeholders was related to MP; however, a stakeholder may be differently placed in sub-CLDs in different regional contexts. The strength of the linkages among the stakeholders is not different based on the model or qualitative nature of the study. However, future empirical studies can include the weightage given to the linkages to establish the critical role of the stakeholders.

The interviews were conducted with operational and field stakeholders that might not capture the complete information about challenges and innovations that are highly localized and are still unnoticed. Future studies can be conducted independently by further sub-categorization of regions. The responses of interviewees might contain unavoidable individual biases. Another limitation is that the CLDs were constructed considering “vaccination doses administered” as a key factor. A demography-based study could be conducted separately to determine vaccination coverage according to gender, rural–urban divide, and socio-economic status. This might include additional and different stakeholders and could highlight the psychological impact post-vaccination. Another limitation of the CLD model is that no exogenous factors have been considered, for instance, impact due to natural calamities such as flooding, which occurred in some districts in MP.

## Conclusion

COVID-19 vaccination campaigns are complex initiatives with many new challenges vis-à-vis routine immunization. Experience and expertize of various stakeholders supported by public participation, real-time support, guidance, data analytics, and training remained crucial for implementing COVID-19 vaccination campaigns. At the same time, the stakeholders were more motivated by their compassion for others, passion for their work, and commitment to contributing toward saving lives and serving society. The study has highlighted an overall causal-loop diagram for the vaccination campaign in MP that depicts the cause-and-effect relationship among various stakeholders. It also highlights the challenges and innovations to improve vaccination coverage even when resources are not adequate. Staff involved in the campaign went beyond their duties and contributed immensely to improving vaccination coverage in MP. The system dynamics model will benefit governments and can be used to devise strategies as best practices for vaccination campaigns. This can also assist policymakers in making informed decisions by considering relationships among stakeholders. At the international level, the findings highlight the current systems in one state in India, its challenges, and how they were resolved through policies, initiatives, and innovations. Governments can assess local or region-specific innovations and replicate them in regions with similar challenges.

Key Questions

1. What are the key drivers of Covid Vaccination Campaigns?
2. What are the interlinkages for the stakeholders of the Vaccination Campaigns?

## Supplementary Information

Below is the link to the electronic supplementary material.

Supplementary file1 (PDF 140 kb)

Supplementary file2 (PDF 124 kb)

Supplementary file3 (PDF 107 kb)

## Footnotes

1 Variables and stakeholders are used interchangeably.

2 In 2015, the Ministry of Health and Family Welfare, Government of India introduced the Electronic Vaccine Intelligence Network (eVIN) to strengthen the Universal Immunization Programme (UIP) of the National Health Mission (NHM). eVIN is an integrated package of people, process, and product combining IT infrastructure and trained human resource to enable real-time monitoring of the stock and storage temperature of the vaccines kept in multiple locations across the country. IT infrastructure consists of software and SIM-enabled temperature loggers (product) specially designed to improve vaccine stock keeping practices and temperature monitoring (processes) across eVIN states. https://www.nhp.gov.in/electronic-vaccine-intelligence-network(evin)_pg.

3 Based on the numbers of registered beneficiaries and the priority accorded, vaccination sessions were planned with the considerations of “one session for 100 beneficiaries” (MoHFW).

4 “Community rumors” should not be restricted to vaccine hesitancy as it includes misinformation related to AEFI including death and fear of getting COVID-19 in crowded settings from poor session planning.

5 The main programmatic components of the NHM include health system strengthening in rural and urban areas-reproductive-maternal-neonatal-child and adolescent health, and communicable and non-communicable diseases. The NHM envisages the achievement of universal access to equitable, affordable, and quality health care services that are accountable and responsive to people’s needs.

6 Corona Volunteers educated people and raised awareness about the disease, preventive measures, and safety protocols through folk songs, paintings, posters, and slogans.

7 Development partners were supported by donor resources from USAID, GAVI, the ADB, the WB, BMGF, and others with COVID-19 and non-COVID-19 support.
