# Supplementary material for: Modeling a COVID-19 Vaccination Campaign in the State of Madhya Pradesh, India
Source: Glob J Flex Syst Manag. 2022 Dec 14;24(1):143–61. doi: 10.1007/s40171-022-00326-9 (PMC9750055; doi:10.1007/s40171-022-00326-9)
Supplement: Supplementary file 1 — Supplementary file1 (PDF 140 kb) [file 40171_2022_326_MOESM1_ESM.pdf]

Appendix 1.

Table A1. Roles and Responsibilities of Stakeholders

S.No.	Stakeholder	Description and predominant role/responsibilities	Sub-CLD Group	Role in CLD
1	Central Government	Government of India (GoI) and its administration responsible for centralized policy level decisions such as vaccine allocation to the states, development of CoWIN App		Stakeholder
2	State Government	Government of Madhya Pradesh (GoMP) constituting state administration machinery. Responsible for policy decisions in various domains		Stakeholder
3	Vaccines Allocation methodology	Vaccine allocation from centre to state and also vaccine distribution among various districts by GoMP		Process
4	Session planning	Daily multiple vaccination sessions at vaccination sites planned and organized		Process
5	Financial planning	Planning done for allocation of funds for COVID-19 vaccination campaign such as infrastructure enhancement and cold chain storage equipment		Process
6	State leadership	Individual officials responsible for decisions in respective domains at state level such as State Enhancement Programme Immunization Officer (SEPIO) and State Cold Chain Officer (SCCO)	Policy	Stakeholder
7	District leadership	Individual officials responsible for decisions in respective domains at state level such as District Immunization Officer (DIO) and Chief Medical and Health Officer (CMHO)		Stakeholder
8	Control rooms & Task forces	COVID-19 Command Centre (CCC) as a control room and task forces for immunization at state, district and block level		Stakeholder
9	Divisional Coordinators	Coordinator for immunization at divisional level (state of MP is divided into seven broad regional divisions)		Stakeholder
10	Development partners	External and global agencies such as CHAI, JSI, KPMG, UNICEF, UNDP, WHO		Stakeholder
11	COVID-19 situation	COVID-19 pandemic outbreak and various parameter to watch its severity and impact on country, states, economy, and people		Ecosystem
12	Political will	Intent of state government, its administration, and leadership		Ecosystem
13	Vaccines Distribution & Storage	Alternate vaccine delivery (AVD) system responsible for vaccine delivery		Process
14	AEFI Management	Resources responsible to deal adverse effect following immunization (AEFI) related issues such as medical help, AEFI kits, grievances, monitoring etc.		Process
15	Cold chain storage & readiness	Cold chain supply-chain for vaccine storage and its stock management	Infrastructure (Physical, HR, Technological)	Process
16	Transport infrastructure, Distribution network, & AVD	Transportation and logistics infrastructure		Infrastructure
17	Vaccines, Syringes and other session logistics	Stock of vaccines, syringes, and other session logistics		Infrastructure
18	AEFI kits	AEFI dedicated kit containing various medical components required to treat AEFI cases		Infrastructure

19	Session site readiness	All the components, human resource, technical and physical infrastructure required for functioning of vaccination sites		Process
20	Session network	Technological infrastructure like internet connection required for functioning of the technological Apps such as CoWIN		Infrastructure
21	Vaccinator - ANMs	Auxiliary Nursing Midwife (ANM) who administer COVID-19 vaccine to the beneficiaries		Stakeholder
22	Vaccination team	Comprises of five members – vaccinator officer (doctors, staff nurse, pharmacist, ANMs); vaccination officer 1 (one person from police, home guard, civil defence etc. to check the registration of beneficiary at entry and ensure regulated entry at vaccination site); vaccination officer 2 (verifier to authenticate or verify the documents); vaccination officer 3 & 4 (two support staff for crowd management and will provide IEC message, support vaccinator and vaccination team)		Stakeholder
23	Cold chain workers	State cold chain officer (SCCO), vaccine and cold chain handlers (VCCH/CCH), cold chain technicians (CCT), store in-charge etc.		Stakeholder
24	ASHA & Anganwadi	Accredited Social Health Activist (ASHA) and Anganwadi workers (AWWs) for effective social and community mobilization are included in frontline health workers (FHW)		Stakeholder
25	Software - CoWIN, G-sheets	CoWIN App is a mobile and web portal to capture details and manage parameters such as vaccination session creation, vaccination booking, demographic details of beneficiaries, doses administered, vaccination certificate download and many more functionalities. Google Sheets (G-sheets) used as a parallel for data entry during initial phase of vaccination campaign		Infrastructure
26	Private health sector	Include private hospitals, nursing homes, clinics, diagnostic laboratories etc.		Stakeholder
27	NGOs	Non-governmental organizations (NGOs)		Stakeholder
28	Industrial bodies	Various industrial bodies to support infrastructure for the vaccination campaign or for community mobilization		Stakeholder
29	Government Inter-departments	Various government departments and ministries such as women and child development, health department, AYUSH, urban and rural development, state police department, defence, minority affairs, tribal affairs, information and technology, railways, education, transportation, sports and youth, revenue, etc.		Stakeholder
30	Mobilisation plan	Plan for social mobilization and community engagement	Communication and Information	Information
31	IEC and communication strategy	Information, education and communication (IEC) strategy to address four key areas such as information on the new COVID-19 vaccine, vaccine hesitancy, vaccine eagerness, COVID-19 appropriate behaviours (CABs)		Process
32	Campaign strategy and adaptability	Strategy for sharing relevant information on the vaccination campaign (pre-vaccine launch and during campaign)		Process
33	RKSK Sathiya	Rashtriya Kishor Swasthya Karyakram (RKSK) Sathiya are adolescent (age group of 10-19 years) volunteers under the RKSK programme launched by the MoHFW in 2014. It’s aim is to meet million teenagers with emphasis on marginalized and underserved communities to make informed and responsible decisions related to their health and well-being		Stakeholder
34	Community Mobilisers	Reputed and trusted individuals, heads of villages, religious heads, retired government officials such as teachers etc.		Stakeholder

35	Panchayats, Medical Colleges	Members of panchayat such as heads, members, ‘*sachiv*’ (secretary) and medical colleges		Stakeholder
36	Youth influencers	Young influencers such as social media influencers, celebrities, sports person		Stakeholder
37	Education institutes	Educational institutes such as schools and colleges		Stakeholder
38	Tribal & Forest department	Departments under Ministry of Tribal Affairs and State Forest Department		Stakeholder
39	Vaccine hesitancy	Refusal or reluctance shown by an individual towards vaccines due to various reasons despite the availability of vaccine services		Information
40	Community rumours	Misinformation or disinformation circulating related to COVID-19 vaccines among communities and individuals		Information
41	Communication & Media	Department of information and publicity and state media agencies at state and district levels, issue of press releases to provide relevant information, timely and clear information to avoid rumours and misconception		Stakeholder
42	Review & Monitoring	Supervision by different stakeholders to review and monitor different aspects related to reporting, data, feedback, etc.	Monitoring and Management	Information
43	Security & Police	State police department and security forces		Stakeholder
44	Data analytics	Technical and human resource for analysing data related to COVID-19 vaccination campaign		Infrastructure
45	Training Department	Different agencies, development partners, state department imparting training to various stakeholders at state and district levels		Stakeholder
46	Tele-calling unit	State tele-calling helpline staff at COVID-19 Command Centre (CCC) and at national level		Stakeholder
47	COVID-19 Volunteers	Citizens of MP who volunteered to work for the COVID-19 vaccination campaign		Stakeholder
