# Supplementary material for: Modeling a COVID-19 Vaccination Campaign in the State of Madhya Pradesh, India
Source: Glob J Flex Syst Manag. 2022 Dec 14;24(1):143–61. doi: 10.1007/s40171-022-00326-9 (PMC9750055; doi:10.1007/s40171-022-00326-9)
Supplement: Supplementary file 2 — Supplementary file2 (PDF 124 kb) [file 40171_2022_326_MOESM2_ESM.pdf]

Appendix 2.

Table A2. Questions for semi-structured interviews

Interview Questions (English)

1. How do you see the vaccination programme in MP in terms of successful implementation?
2. How officials' (stakeholder) role is critical in COVID-19 vaccination process?
3. How do you think that technology intervention has helped to improve the COVID-19 Vaccination programme?
4. What are the state specific challenges faced and solutions implemented in the COVID-19 vaccination programme in MP? What was unique in the solutions?
5. What are the success stories/best practices that contributed in the improvement in the COVID-19 vaccination coverage?
6. Can you comment on how people in MP reacted to vaccine hesitancy misinformation?
7. Can you also comment on how people in MP responded to IEC and stakeholder mobilization?
8. How much the situation has been improved during last 3-4 months? (In terms of community mobilization)
9. What are the foreseen challenges? Any other suggestions or ideas to deal with such challenges.

साक्षात्कार के प्रश्न (हिंदी)

1. मध्य प्रदेश में टीकाकरण कार्यक्रम को सफल क्रियान्वयन के संदर्भ में आप किस प्रकार देखते हैं?
2. COVID-19 टीकाकरण प्रक्रिया में अधिकारियों (हितधारक) की भूमिका कैसे महत्वपूर्ण है?
3. आप कैसे सोचते हैं कि प्रौद्योगिकी हस्तक्षेप ने COVID-19 टीकाकरण कार्यक्रम को बेहतर बनाने में मदद की है?
4. मध्य प्रदेश में COVID-19 टीकाकरण कार्यक्रम में राज्य की विशिष्ट चुनौतियों का सामना और समाधान क्या हैं? समाधानों में अद्वितीय/नया क्या था?
5. ऐसी कौन सी सफलता की घटनाएं/सर्वोत्तम प्रथाएं हैं जिन्होंने COVID-19 टीकाकरण कवरेज में सुधार में योगदान दिया है?
6. क्या आप टिप्पणी कर सकते हैं कि मध्य प्रदेश के लोगों ने COVID-19 टीके लगाने में हिचकिचाहट वाली गलत सूचना पर कैसी प्रतिक्रिया दी?
7. क्या आप इस पर भी टिप्पणी कर सकते हैं कि मध्य प्रदेश के लोगों ने COVID-19 आईईसी (IEC) और हितधारक जुटाव (Community mobilization) के प्रति कैसी प्रतिक्रिया व्यक्त की?
8. पिछले 3-4 महीनों के दौरान स्थिति में कितना सुधार हुआ है? (समुदाय जुटाव के संदर्भ में)
9. COVID-19 से संबंधित भविष्य में क्या चुनौतियाँ हैं, क्या आप देखते हैं? कोई अन्य सुझाव या विचार जो इन चुनौतियों से निपट सकते हैं।
