# Supplementary material for: Modeling a COVID-19 Vaccination Campaign in the State of Madhya Pradesh, India
Source: Glob J Flex Syst Manag. 2022 Dec 14;24(1):143–61. doi: 10.1007/s40171-022-00326-9 (PMC9750055; doi:10.1007/s40171-022-00326-9)
Supplement: Supplementary file 3 — Supplementary file3 (PDF 107 kb) [file 40171_2022_326_MOESM3_ESM.pdf]

Appendix 3.

Table A3. Summary of findings of sub-CLDs

(stakeholders under each sub-CLD are given in Annexure 3 and stakeholders who were interviewed are provided in Table 1)

S.No.	Sub-CLD Group	Input	Output	Impact of CLD
1	Policy	Various stakeholders involved and responsible for the policy level decisions and framing strategies, planning for infrastructure and resources were interviewed. The stakeholders in CLD includes state and district leadership and administration, development partners and their role in planning and strategy related to the COVID-19 vaccination campaign	Based on the responses from the respondents, impact of policy level decisions and flow of instructions, relationship among stakeholders, implementation of strategy on vaccination doses administered was established	This CLD highlights key constituents responsible for policy decisions. Policymakers can observe relationship among different administration constituents and hierarchy of flow of instructions and how decisions at the top level impacts vaccination doses administered
2	Infrastructure (Physical, HR, Technological)	Stakeholders involved and responsible for human, physical and technological resources were interviewed. It also included processes that resulted in infrastructure enhancement and capacity building resources such as transport and distribution network, cold chain storage, vaccination sites, vaccination team, private health sector, session network, etc.	Based on responses from the respondents at state and district levels, impact of infrastructure development on vaccination doses administered was validated and relationship among stakeholders was established	This CLD highlights importance of preparedness related to COVID-19 vaccination campaign infrastructure. Variables that played substantial role in improving vaccination. Policymakers can find key stakeholders responsible and relationship among them
3	Communication & Information	Stakeholders involved and responsible for flow of relevant information related to new information related to COVID-19 vaccine, vaccination, and community mobilization to reduce vaccine hesitancy. It includes vaccination team, ANMs, AWWs, community mobilizers, youth influencers, RKSK sathiya etc.	Based on the responses from the respondents, impact of relevant information sharing and clarifying community rumours helped in reducing vaccine hesitancy that helped improving vaccination doses administered. Relationship between community rumours and vaccine hesitancy was also established	This CLD highlights flow of communication among different stakeholders responsible for community mobilization and communicating IEC strategy. Communication and media planning department can observe constituents that played substantial role to address vaccine hesitancy and community mobilization
4	Monitoring & Management	Stakeholders involved and responsible for the management of public order, crowd management, cold chain supply, review and monitoring of data, software, and data analytics were interviewed. It includes task force and control rooms and how they managed, monitored and supervised the reporting, data, grievances etc.	Based on responses from respondents, impact of monitoring and management of different crucial aspects in the process of vaccination on vaccination doses administered was established. Flow of feedback and data back up towards hierarchy can help in data analytics and grievances redressal	This CLD highlights importance of diverse components in smooth functioning of vaccination sites, cold chain storage, etc. deployed in vaccination campaign. Government inter-department collaboration can be observed that can cater to both aspects i.e. front-end and back-end management
